# A Mixed-Methods Process Evaluation: Integrating Depression Treatment Into HIV Care in Malawi

**DOI:** 10.9745/GHSP-D-20-00607

**Published:** 2021-09-30

**Authors:** Melissa A. Stockton, Caroline E. Minnick, Kazione Kulisewa, Steven M. Mphonda, Mina C. Hosseinipour, Bradley N. Gaynes, Joanna Maselko, Audrey E. Pettifor, Vivian Go, Michael Udedi, Brian W. Pence

**Affiliations:** a Epidemiology Department, Gillings School of Global Public Health, University of North Carolina at Chapel Hill, Chapel Hill, NC, USA.; b University of North Carolina Project-Malawi, Tidziwe Centre, Lilongwe, Malawi.; c Department of Mental Health, College of Medicine, University of Malawi, Blantyre, Malawi.; d Department of Psychiatry, University of North Carolina at Chapel Hill School of Medicine, Chapel Hill, NC, USA.; e Noncommunicable Diseases and Mental Health Unit, Ministry of Health, Lilongwe, Malawi.

## Abstract

**Background:**

Depression is highly prevalent among people living with HIV in Malawi. Depression can undermine engagement in HIV care and worsen HIV morbidity and mortality. The Ministry of Health integrated a pilot depression management program into HIV care at 2 clinics. This program included a measurement-based care protocol for prescribing antidepressants and an adapted Friendship Bench psychotherapy protocol for providing problem solving. Early evaluations indicated successful integration of the initial stages of training and depression screening, diagnosis, and treatment initiation. This follow-up mixed-method investigation contextualizes our previous findings and shares insights from the implementation experience.

**Methods:**

We conducted a mixed-methods process evaluation drawing on both patient clinical data and qualitative interviews with patients and clinic staff. We focus on the following implementation outcomes: fidelity, acceptability, and sustainability.

**Results:**

Although fidelity to depression screening and treatment initiation was high, fidelity to the follow-up treatment protocol was poor. Antidepressants and problem-solving therapy were acceptable to patients, but clinic staff found delivering treatment challenging given constrained human resources and infrastructure. The program was not sustained after the project. Key identified needs included substantial support to supervise the implementation of the program, continue to build and maintain the capacity of providers, integrate the program into the electronic medical records system, and ensure the availability of Friendship Bench counselors.

**Conclusions:**

Although initial steps were successful, sustained integration of this depression treatment program into HIV care in this setting met greater challenges. Implementation science studies that support both implementation and evaluation should recognize the potential for clinical implementers to rely on evaluation staff for clinical support and consider distancing evaluation staff from the actual program implementation. Further research is needed to test enhanced implementation strategies for integrating evidence-based mental health interventions into existing health care systems in a sustainable fashion, particularly in low-resource settings.

## Abstract (precis)

Effectively integrating depression treatment into HIV care in low-resource settings will require substantially investing in program supervision, building and maintaining the capacity of providers, integrating into existing electronic medical records systems, and ensuring the availability of psychotherapy counselors.

## Teaser

**Key Findings:**

- Early evaluations of integrating a depression management program using antidepressants and psychotherapy into HIV clinics in Malawi found that depression screening, diagnosis, and treatment initiation were successfully integrated.
- A follow-up evaluation found that treatment over time was not delivered as intended, clinic staff had mixed attitudes regarding program acceptability, and the program was not sustained.
- Antidepressants and problem-solving therapy were acceptable evidence-based depression treatments for patients, but clinic staff found delivering these treatments challenging given constrained human resources and infrastructure.
- Delivery challenges included difficulties identifying and reassessing patients, negative attitudes, lack of integration into the electronic medical records system, medication stock-outs, staff and space availability, cost of transport, generally low retention, and reliance on study staff.

**Key Implications:**

- Program managers need to invest in program supervision, build and maintain the capacity of providers, integrate into existing electronic medical records systems, and ensure the availability of psychotherapy counselors to more effectively integrate depression treatment into HIV care in these types of settings.

## INTRODUCTION

The burden of depression is high among people living with HIV, particularly in sub-Saharan Africa (SSA).^1^ Depression hinders engagement in HIV care and antiretroviral therapy (ART) adherence, which ultimately predict HIV clinical morbidity and mortality.^1^^–^^6^ Despite the deleterious impact of untreated depression, low levels of investment in mental health care persist across the globe and especially in resource-limited settings such as SSA.^7^ Fortunately, interventions to address the burden of depression among people living with HIV are being developed,^8^^,^^9^ with several specifically for SSA or other settings with limited psychiatric infrastructure and human resources.^10^^–^^15^

To address the burden of depression among people living with HIV, the Malawi Ministry of Health (MOH) implemented a pilot task-shifting program that integrated depression management into ART initiation at 2 clinics in Lilongwe, Malawi.^16^^,^^17^ As part of this program, existing clinic-based staff provided 2 evidence-based depression treatment interventions: measurement-based care (MBC) antidepressant prescription^10^^,^^15^^,^^18^ and the Friendship Bench problem-solving therapy.^14^^,^^19^ Early assessments of program delivery demonstrated the feasibility of integrating depression screening into ART initiation and starting patients on antidepressants or Friends Bench therapy.^17^^,^^20^ Despite the proven efficacy of these treatment models, the initial program evaluation found that the treatment program did not improve retention, viral suppression, or depression remission.^20^

The ultimate impact of health innovations depends not only on their effectiveness, but also on their reach and the extent to which they are properly adopted, implemented, and maintained over time.^21^ In this manuscript, we first describe how the program was designed. We then present a mixed-methods process evaluation investigating the extent to which the treatment program was delivered with fidelity at treatment initiation and over time, deemed acceptable by providers and patients, and sustained after the conclusion of the program evaluation. This mixed-method investigation allows us to contextualize our previous findings^17^^,^^20^ and share insights from the implementation experience.

The impact of health innovations depends not only on their effectiveness, but also how well they are adopted, implemented, and maintained over time.

## PROGRAM OVERVIEW

The pilot program integrated depression screening and treatment into ART initiation at 2 semi-urban primary-level health facilities in Lilongwe, Malawi. The intervention launched at Clinic A in November 2017 and at Clinic B in April 2018. These public facilities provide services free of charge and encompass an ART clinic or department. Approximately 25 nurses and clinicians provide ART services at each site, although staff turnover is high. Only 1 facility employs a psychiatric nurse who does not regularly offer psychiatric services. Private mental health care specialists irregularly offer mental health clinic days at the facilities. Otherwise, patients can be referred to the psychiatric department at the district hospital. Further details on the health facilities are available in previous publications.^17^

**FIGURE 1:**
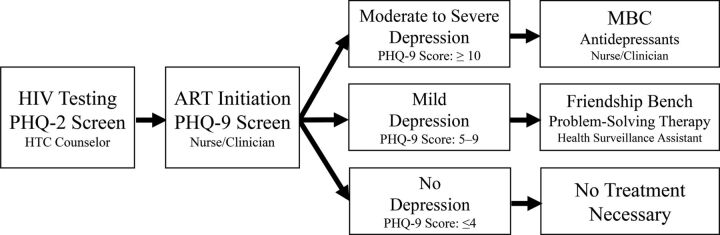
Algorithm Used by Providers for Screening and Managing Patients With HIV for Depression Who Were Newly Initiating Antiretroviral Therapy, Malawi Abbreviations: ART, antiretroviral therapy; HTC, HIV testing and counseling; MBC, measurement-based care; PHQ, Patient Health Questionnaire.

HIV testing and counseling (HTC) counselors and ART providers were trained to conduct depression screening of patients when they were newly initiating ART by using the Patient Health Questionnaire-9 (PHQ-9), a 9-item screening questionnaire that has been widely used in the region.^22^^–^^26^ HTC counselors screened patients who tested positive for HIV with the first 2 questions of the PHQ-9 (the Patient Health Questionnaire-2 [PHQ-2]) that screen for depressed mood and anhedonia. ART providers completed the remaining 7 questions of the PHQ-9 with any patients who endorsed either of the symptoms captured by the PHQ-2 (Figure 1).

HTC counselors and ART providers were trained to conduct depression screening of patients newly receiving ART and to refer them for antidepressants or counseling as needed.

For patients with moderate to severe depression (PHQ-9 score ≥10), ART providers were trained to treat them with antidepressants (amitriptyline or fluoxetine) using MBC (Figure 1). MBC is a resource-efficient, task-sharing model for prescribing antidepressant management in nonpsychiatric settings that has demonstrated safety, feasibility, and acceptability when adapted for HIV care and delivered by nonspecialists in Cameroon, Tanzania, and Uganda.^10^^,^^15^^,^^18^ The pharmacies at the health facilities were instructed to stock amitriptyline and fluoxetine, 2 antidepressants that are considered “essential medicines” under Malawi’s Essential Health Package and are meant to be freely available for patients at health facilities.^27^^,^^28^ ART providers were instructed to discuss the available antidepressants with the patient and jointly decide on the patient’s best option. When patients returned for ART care, ART providers were then meant to reassess patients’ depressive symptoms, evaluate their response to the depression treatment, and prescribe antidepressants as necessary. The study staff hung posters in the ART clinic rooms that provided an overview of the MBC protocol and detailed how to use PHQ-9 scores and adverse-effect tolerability to guide changes in dosage or type of antidepressant (Supplementary Material 1). A standard course of antidepressants should consist of at least 3 consecutive months of antidepressant prescription with dosage adjustments as necessary.

For patients with mild depression (PHQ-9 score 5–9), ART providers were trained to refer them to clinic-based community health workers (called health surveillance assistants [HSAs]) who were trained to provide Friendship Bench problem-solving therapy (Figure 1). Friendship Bench therapy is an adaption of problem-solving therapy or patient-centered counseling developed in Zimbabwe that teaches patients how to identify triggers and effectively manage stressful life events by learning or reactivating problem-solving skills.^14^^,^^19^ The Friendship Bench protocol planned for referred patients to receive at least 6 counseling sessions, with adequate treatment comprising at least 6 sessions within the first 6 months. While Friendship Bench counselors were instructed to encourage patients to return weekly for Friendship Bench therapy, patients set their own appointments in line with the protocol’s patient-centered approach. While 8 HSAs at each site were trained in the Friendship Bench protocol, these HSAs continued to be responsible for fulfilling their previous duties. The responsibilities of a clinic-based HSA vary but may include activities such as following up with patients who have dropped out of care or managing community outreach vaccination campaigns. The study also employed 1 Friendship Bench counselor at each site who attended the same Friendship Bench training as the HSAs. The study-employed counselors were meant to provide backup support for the HSAs trained in the Friendship Bench protocol. Additionally, to address counseling space limitations, the study built covered shelters at both sites as a dedicated space for the Friendship Bench therapy.

**Figure:**
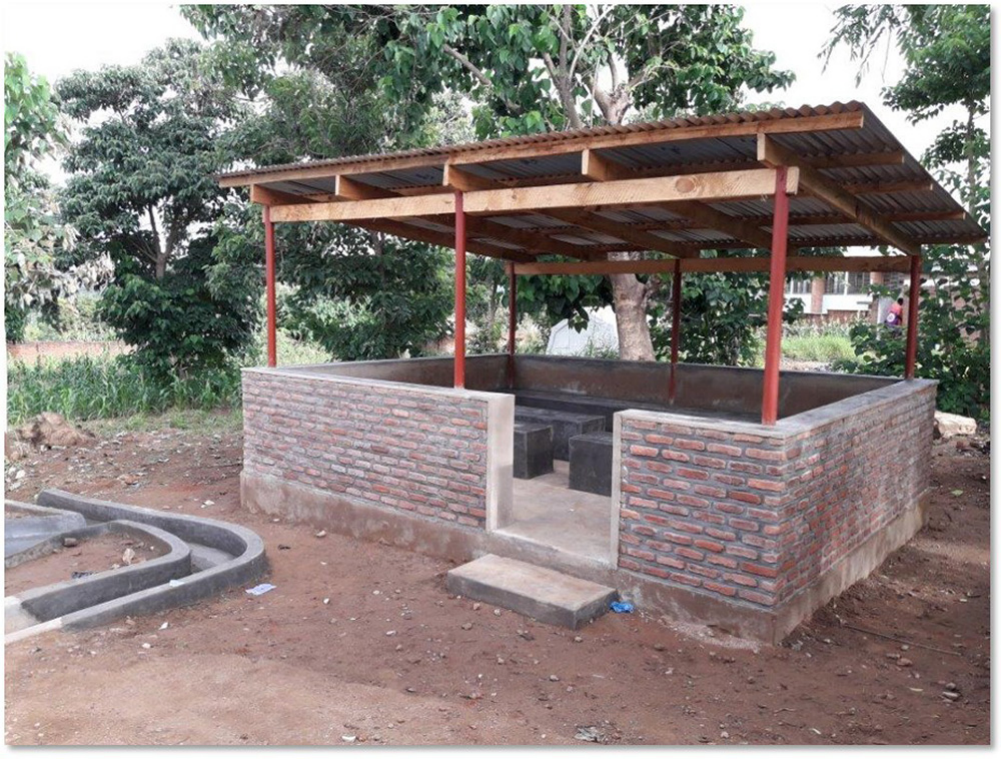
A shelter dedicated for Friendship Bench therapy that was built at one of the program sites in Lilongwe, Malawi. ©2018 Steven Mphonda/University of North Carolina Project-Malawi.

Throughout the program implementation, the study coordinator held bimonthly meetings at each site to support clinic staff and leadership deliver the program. However, the study staff were otherwise not meant to assist in the provision of daily clinical care and were only meant to consent patients and abstract their clinical data. Before the conclusion of the program evaluation, the study coordinator worked with the health facility staff and leadership to transition ownership of the program entirely to the health facilities. As such, the HIV providers were to continue screening and treating patients according to the Friendship Bench and MBC protocols after the conclusion of the study evaluation. Previous publications provide further details on the program design,^16^ implementation,^17^ and impact.^20^

Before the program evaluation concluded, ownership of the program transitioned to the health facilities.

## METHODS

### Clinical Data

After the launch of the intervention, all nonpregnant adults newly initiating ART were exposed to the program and eligible to participate in the program evaluation. Between April 2017 and November 2018 at Clinic A and between April 2018 and November 2018 at Clinic B, study staff approached potential participants during the ART initiation process to invite them to allow their clinical data to be used in the program evaluation. Study staff abstracted sociodemographic information as well as data on depression and HIV care from consenting participants’ clinical records over a 13-month period, starting at ART initiation. Again, further details on enrollment in the program evaluation, clinical data abstraction, and the measures captured have been previously published.^16^^,^^20^ Only participants with elevated depressive symptoms (PHQ-9 ≥5) at ART initiation are included in this mixed-methods process evaluation. All statistical analyses were performed using STATA IC 14.

### Qualitative Data

In-depth interviews were conducted between June and December 2018 with the following objectives: (1) explore providers’ and patients’ attitudes toward and experiences with the depression treatment intervention; (2) understand barriers and facilitators to integrating depression treatment into HIV care; and (3) prepare for the long-term sustainability of the program after the conclusion of the program evaluation. The qualitative interviews were conducted with a convenience sample of patients returning for ART care. Patients were eligible to participate if they had previously been identified with depression at ART initiation and either referred to the Friendship Bench or prescribed antidepressants. Additionally, a convenience sample of ART providers, Friendship Bench counselors, and clinic leadership were also interviewed. Before data collection, study staff met with the administrators at both health centers and drew up a list of staff. The study coordinator or interviewer approached staff and leadership at the clinic to schedule interviews. The study staff identified eligible patients returning for ART services and invited these patients to participate. An effort was made to interview a range of men and women because prior research showed that attitudes toward depression and the provision of mental health care may vary by the gender of both providers and patients.^29^^–^^31^ The research team developed semistructured interview guides based on the study objectives. Interviews were conducted in either Chichewa (the local language) or English, based on participants’ preference, by a Malawian woman with a background in qualitative research and HIV care services. All interviews were held at the respective health facility in a private location. The interviews were audio-recorded, transcribed, and translated into English (if necessary). The research team reviewed transcripts as they became available and provided feedback to the interviewer throughout the data collection process.

Interviews were conducted to explore attitudes towards the depression treatment intervention and the barriers and facilitators to integrating the treatment into HIV care.

After reviewing the transcripts, the author (MS) drafted a thematic codebook that would address the study objectives and capture emerging themes evident from her initial review of the transcripts.^32^^,^^33^ MS then met with research team members to review and finalize the codebook. Two coders (MS and CM) coded a subset of the same transcripts and resolved any discrepancies to ensure consistency in their use of the codes using NVivo (Version 12). Coding was treated as an iterative process, and the coders met several times throughout to discuss the addition, definition, and appropriate use of the codes that emerged from the data. Upon completion of coding, the coders executed queries in NVivo and reviewed coded data related to the key aspects of program implementation, depression treatment initiation and treatment over time, acceptability, and sustainability. The coders created detailed summaries relevant to each code, took inventory of the emerging principal themes, and observed any variation across participant groups. While synthesizing these findings, MS met with the study team to ensure accurate interpretation of the interviews.

### Implementation Outcomes

We defined the implementation outcomes in line with Proctor and colleagues’ conceptualization of implementation science research outcomes^34^ and Carroll and colleagues’ framework for implementation fidelity.^35^ Fidelity is the degree to which this program was implemented as intended or the adherence to the program protocol. Acceptability is the perception among stakeholders and consumers—in this case, health facility staff, leadership, and patients—that the program is agreeable or satisfactory based on their knowledge, experience, and comfort with the program’s content and complexity. Sustainability is defined as the extent to which the program is maintained within the health facilities’ ongoing operations after the conclusion of the program evaluation. We assess each of these outcomes in the following manner.

Implementation outcomes were defined by fidelity to the program protocol, acceptability to stakeholders and consumers, and sustainability of the program itself.

#### Fidelity

We examined adherence to the program treatment protocol in terms of content, coverage, frequency, and duration^35^ by using clinical data to determine whether patients initiated the correct depression treatment (content) based on their depressive severity (coverage) and whether these patients continued to attend Friendship Bench therapy sessions or receive antidepressants (frequency) over their first 6 months in care (duration). Data from the qualitative interviews explain and contextualize the quantitative fidelity measures.

#### Acceptability

We used data from the qualitative interviews to explore participants’ views toward treatment delivery and its impact on patient outcomes. We specifically considered providers’ comfort with the depression treatment options, their opinions about the complexity of the program, and structural impediments to program delivery. Additionally, patients’ and providers’ experiences with the program’s effect on patient health outcomes were also reviewed.

#### Sustainability

We examined screening rates soon after the study stopped enrolling participants in the program evaluation (from February to May 2019) and then again in December 2019. We also used discussions with clinic staff and leadership about maintaining the program to help understand these findings.

### Ethical Considerations

We obtained approval from both the Malawi MOH’s National Health Science Research Committee (NHSRC) institutional review board (IRB) and the Biomedical IRB of the University of North Carolina at Chapel Hill. All participants who agreed to participate in the study provided informed consent. Qualitative study participants were given a travel reimbursement equivalent of 10 USD (7,000 MK).

## RESULTS

We first describe the characteristics of the study participants and then assess fidelity, acceptability, and sustainability. Fidelity was examined at treatment initiation and over time for both the MBC protocols and Friendship Bench protocols. Of note, themes presented in the fidelity section may reflect participants’ comfort with the program and its overall complexity, and these factors may overlap with the program’s acceptability.

### Participant Characteristics

Of the 936 patients who enrolled in the program after the launch of the intervention, 211 were depressed at the time of ART initiation (Table 1). A convenience sample of 14 of these 211 patients were interviewed. Of these 14 patients, 11 were prescribed antidepressants and 3 started Friendship Bench therapy at ART initiation. In light of the delayed launch at Clinic B, a small number of participants had depression at Clinic B; thus all of the interviewed patients were from Clinic A. Twelve clinic staff were also interviewed.

**TABLE 1.** Characteristics of Participants With Depression at the Time of ART Initiation

	Clinical Data	Qualitative Data	
	Patients (N=211)	Patients (N=14)	Providers (N=12)
Sex, no.			
Female	89	6	7
Male	122	8	5
Age^a^, mean (range)	34.0 (19–65)	36.1 (23–47)	34.2 (26–46)
Health facility, no.			
Clinic A	116	14	6
Clinic B	174	0	6
Marital status^b^, no.			
Married	n/a	6	n/a
Single	n/a	0	n/a
Separated	n/a	7	n/a
Employment, no.			
Employed	n/a	12	n/a
Self-employed	n/a	0	n/a
Unemployed	n/a	1	n/a
Position, no.			
ART provider	n/a	n/a	6
Friendship Bench counselor	n/a	n/a	4
Leadership	n/a	n/a	2
Professional experience			
Years at clinic, mean (range)	n/a	n/a	4.9 (0.7–11)
Years as clinician, mean (range)	n/a	n/a	8.7 (4–15)

Abbreviations: ART, antiretroviral therapy; n/a, not applicable.

^a^ Age missing (n=3) and excludes leadership.

^b^ Marital status and employment missing (n=1).

### Fidelity

#### Measurement-Based Care

The clinical data demonstrate that ART providers successfully prescribed antidepressants at a therapeutic dose in nearly all (96%) cases of moderate to severe depression, achieving high fidelity to the MBC protocol (Table 2). Based on the qualitative interviews, ART providers appeared knowledgeable about the depression treatment protocol and how to appropriately triage patients based on their PHQ-9 scores. The interviews suggested that providers deviated from guidance to discuss the antidepressant options with patients and jointly decide on the best antidepressant. It seemed that providers were most comfortable prescribing amitriptyline and the decision around which antidepressant to prescribe was almost entirely governed by what was in stock. As heard from one ART provider:

**TABLE 2.** Initiation of Depression Treatment Prescribed by ART Providers

	Mild Depression (PHQ-9 score 5–9)* N=156, no. (%)	Moderate to Severe Depression (PHQ-9 score ≥10) N=55, no. (%)
Counseling by ART provider	15 (10)	2 (4)
Friendship Bench	134 (86)	0 (0)
Antidepressant	0 (0)	41 (75)
With counseling by ART provider	0 (0)	8 (15)
With Friendship Bench	2 (1)	4 (7)
None^a^	5 (3)	0 (0)
Of those who start antidepressant	N=2	N=53
Type		
Amitriptyline	1 (50)	37 (70)
Fluoxetine	1 (50)	16 (30)
Dose		
Subtherapeutic	0 (0)	0 (0)
Therapeutic	2 (100)	53 (100)

Abbreviations: ART, antiretroviral therapy; PHQ-9, Patient Health Questionnaire-9.

^a^ Includes 4 patients with missing baseline treatment.

*We just prescribe the antidepressants that are in stock at that time, but the first choice is amitriptyline. If amitriptyline is not available, then we give that patient the other antidepressant.* —ART provider

The clinical data demonstrate that ART providers successfully prescribed antidepressants at a therapeutic dose to nearly all patients with moderate to severe depression.

Leadership and ART providers felt that providers were more familiar with amitriptyline and that it was easier for patients to take. Patients take amitriptyline daily, while they initially take fluoxetine every other day.

**FIGURE 2:**
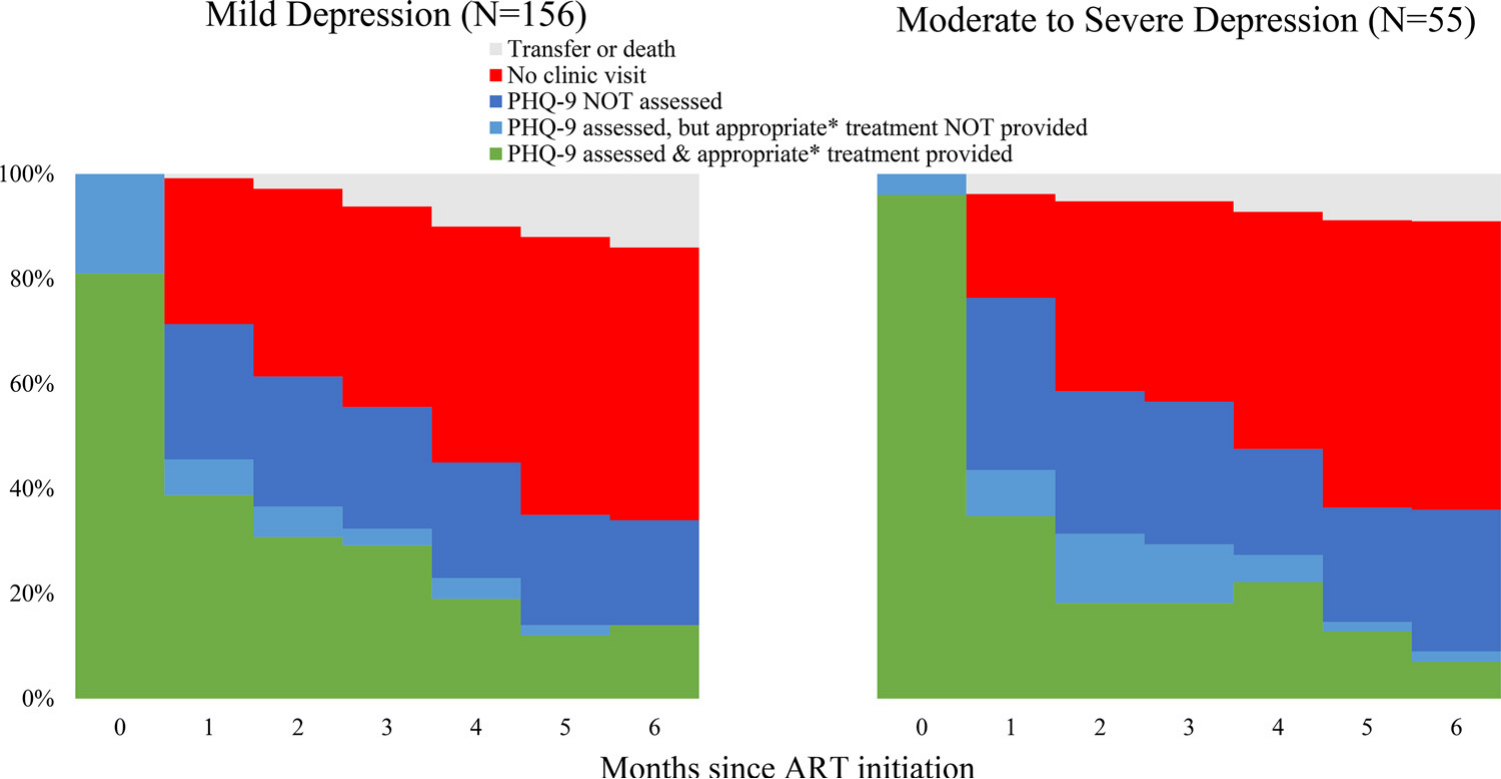
Depression Treatment Engagement of Patients With HIV, by Depressive Severity, Malawi Abbreviations: ART, antiretroviral therapy; PHQ, Patient Health Questionnaire. * Appropriate treatment is operationalized as attending a Friendship Bench session, or having completed 6 Friendship Bench sessions for those with mild depression, or being prescribed antidepressants among those with moderate to severe depression.

The provision of MBC over time was challenging. This challenge was partially due to waning retention over the first 6 months of care. Figure 2 uses clinical data to show that by the first follow-up appointment (month 1) only around three-quarters of participants attended an ART appointment and by month 6 only around a third of participants attended an ART appointment. Among those who did come to follow-up HIV care appointments, providers often failed to reassess these patients with the PHQ-9, which would have guided depression management, or to continue providing appropriate depression treatment to those who were rescreened.

The qualitative interviews helped explain why it was difficult to identify depressed patients returning for care and to reassess those patients for depression. ART providers felt that the process of identifying patients with depression and confirming the diagnosis with the PHQ-9 took a lot of time and significantly added to their workload, especially in light of the general shortage of staff and high patient caseload. For context, study staff timed the PHQ-9 administration, and ART providers took about a minute per question. That said, clinic leadership admitted:

*[ART providers] said they get tired and they don’t even ask the PHQ because they know that “this will take much of my time.” But if we had more personnel more people seeing patients that would have been better*. —Clinic leadership staff person

The qualitative interviews helped explain why it was difficult to identify depressed patients returning for care and to reassess those patients for depression.

Identifying patients was also difficult because the program was not integrated into the electronic medical records (EMR) system. Providers had to look for stickers on the patients’ health passports that signaled they had previously been diagnosed with depression. If providers noticed the sticker, they would then manually search for the patient’s file, reassess the patient with the PHQ-9, and then provide appropriate treatment. It was difficult for providers to determine when a patient no longer needed to be assessed or had completed their depression treatment. One ART provider ventured:

*There wouldn’t be a problem if depression questions were in the electronic system.* —ART provider

As an extension of these issues, ART providers often had a poor attitude toward the program. This phenomenon was summarized by clinic leadership,

*The workload [is problematic] when you are alone in the ART room and you need to screen those people, so sometimes people may be annoyed, [thinking] “I should screen this one, but this will delay me.”* —Clinic leadership staff person

Other interviewed ART providers echoed this sentiment. As a result, ART providers often relied on the study staff to identify patients with depression returning for care, and the accuracy of the follow-up PHQ-9 assessments may have been compromised.

Few of those with moderate to severe depression received antidepressants continuously for the recommended minimum 3 months based on analysis of the clinical data (Table 3). Only 6 participants received at least 3 consecutive months of the same antidepressant. Switching the type of antidepressant prescribed was common; of the 30 individuals who were prescribed antidepressants at least twice, 40% (n=12) changed antidepressants at least once. Providers also failed to increase the dose of antidepressants for any of the patients when indicated by persistent elevated symptoms as dictated by the MBC protocol.

**TABLE 3.** Duration of Antidepressant Provision to Patients With Moderate to Severe Depression at Baseline (N=55)

	Moderate to Severe Depression (PHQ-9 score ≥10), no. (%)
Months of antidepressant prescription	
0	2 (4)
1	23 (42)
2	14 (25)
3	7 (13)
≥4	9 (16)

Abbreviation: PHQ-9, Patient Health Questionaire-9.

The qualitative interviews provided insight into the barriers to achieving the recommended duration of the antidepressant prescription. The main challenge was rooted in identifying patients returning for care, as previously described. While prescribing antidepressants added to ART providers’ workload, providers were much more concerned about the time it took to identify and rescreen patients. When asked specifically about the added workload of prescribing antidepressants, one ART provider responded:

*The issue is just the time to ask the PHQ only, the time to ask the PHQ is what troubles us.* —ART provider

The qualitative interviews provided insight into the barriers to achieving the recommended duration of antidepressant prescription.

Additionally, antidepressant stock-outs were common, as noted by an ART provider:

*It’s very rare whereby you have both [antidepressants] available …, sometimes you find that the one [the patient started taking] is not available so we switch them.* —ART provider

These reports from the clinics show that switches in antidepressant prescription were not due to side effects or lack of effect, but necessitated by stock-outs. Ensuring that staff were properly trained on the MBC protocol was also challenging, due to high staff turnover and/or rotation through clinic departments and staff availability to attend the original trainings or the supervision sessions. Clinic leadership spoke to these needs:

*The training was just one day, so most people were not familiar with the dosages so they need to be reminded here and there. Of course, there are debriefing meetings that are done on Thursdays, but still not everybody attends. So prescribing is difficult just because people forget the dosage of the medication.* —Clinic leadership staff person

This feedback highlights the complexity of MBC and a need for ongoing support and training. From a patient perspective, actually taking antidepressants appeared largely acceptable. While some providers were concerned about the added pill burden, patients mostly found the antidepressants both tolerable and acceptable.

#### Friendship Bench

The clinical data demonstrate that nearly all (86%) of cases of mild depression correctly started the Friendship Bench therapy that same day, achieving high fidelity to the Friendship Bench protocol. However, the study-employed Friendship Bench counselor provided over half of the initial Friendship Bench therapy sessions (Table 2). As noted by one ART provider,

*Most of the time they [Friendship Bench counselors] are in the field, so we refer them to the [study-employed counselor].* —ART provider

This statement suggested that the clinic-based Friendship Bench counselors were often unavailable, resulting in heavy reliance on the program-employed counselor.

The clinical data revealed that although almost all patients with mild depression at ART initiation started the Friendship Bench, few participants received consistent Friendship Bench therapy over their first 6 months in care (Table 4). The original Friendship Bench therapy protocol was designed to be administered over the course of 6 consecutive weekly sessions. None of the patients completed this ideal “full course” in their first 2 months of care. Only 13 patients completed at least 6 Friendship Bench sessions within their first 6 months of care. The study-employed Friendship Bench counselor provided over half of the follow-up sessions.

**TABLE 4.** Duration of Friendship Bench Therapy Provided to Patients With Mild Depression at Baseline (N=156)

	Mild Depression (PHQ-9 Score 5–9),^a^ no. (%)	
	Within the first	
	2 months	6 months
Friendship Bench sessions attended		
0	20 (13)	20 (13)
1	64 (41)	59 (38)
2	32 (21)	19 (12)
3	37 (24)	13 (8)
≥4	3 (2)	45 (29)

Abbreviation: PHQ-9, Patient Health Questionnaire-9.

^a^ Includes 4 patients with missing baseline treatment.

The qualitative interviews help explain the challenges to achieving the recommended number of Friendship Bench sessions. Both patients and providers reported that scheduling weekly Friendship Bench sessions was almost impossible for patients because the cost of transport was prohibitive, clinic wait times were long, and patients would have to take off work. For this reason, patients often chose to schedule their Friendship Bench appointments monthly in conjunction with their ART appointments. As described by one Friendship Bench counselor:

*Some [challenges] are transport … we [Friendship Bench counselors] give dates and ART give their dates. … So the person weighs the cost … Many are prioritizing coming for [ART] medications rather than just coming for the Friendship Bench*.—Friendship Bench counselor

Both patients and providers reported that scheduling weekly Friendship Bench sessions was almost impossible for patients.

Beyond highlighting the complexities of attending follow-up Friendship Bench sessions in light of constrained resources, this quote also hints at a disconnect between the ART and weekly depression care for patients on Friendship Bench therapy.

The same staffing challenges that impeded initiating Friendship Bench therapy were also raised for providing therapy over time. The Friendship Bench counselors were not always available owing to their competing responsibilities as community health workers, and they felt their workload had substantially increased. While the Friendship Bench counselors strove to share the added workload, the clinics often relied heavily on the program-employed Friendship Bench counselor, as previously described. Friendship Bench counselors felt the provision of the Friendship Bench therapy was hampered by the sheer number of patients who defaulted or failed to attend follow-up sessions. They were frustrated by their inability to adequately trace patients in light of phone numbers not working, difficulties locating patients’ residences, and limited funds for airtime or transport.

The qualitative interviews also suggest patient discomfort may have played a role in poor continued engagement in Friendship Bench therapy. Interviewed Friendship Bench counselors believed it was important to develop rapport with patients, create an environment where patients felt comfortable speaking openly, and maintain patient privacy. Patients echoed this sentiment, both as a concern and as something they appreciated about the Friendship Bench therapy. As an extension of this, clinic staff and leadership raised concerns about the availability of confidential spaces. One Friendship Bench counselor noted that other patients were often nearby the Friendship Bench shelters and “when the client sees that, they feel that when we tell them that what we are going to discuss is confidential, we cheat on them because people are passing by.” This suggests that the open, outdoor nature of these shelters may not be conducive to the provision of depression counseling in this setting.

### Acceptability

The qualitative interviews show that nearly all patients, staff, and leadership believed both MBC and the Friendship Bench helped improve patients’ mental health, relieve their depressive symptoms, and prevent risk of suicide. In contrast, the previously published quantitative evaluation found that the treatment program did not demonstrably improve retention in HIV care, viral suppression, or depression remission 6 months after ART initiation.^20^ In the qualitative interviews, ART providers cited patients’ reduced PHQ-9 scores as proof of the effectiveness of both antidepressants and the Friendship Bench. The Friendship Bench counselors attributed the program’s perceived success to its focus on teaching patients to identify their own problems and solutions. Some patients described the specific ways their respective depression treatment helped them. For example, patients believed treatment allowed them to calm their thoughts and to eat and sleep regularly. As heard from one patient taking antidepressants:

*I am able to talk normally and I do not have any anxiety/depression. I am now able to chat with my friends, just like the way life should be.* —Patient

Nearly all patients, staff, and leadership believed screening and treatment helped improve patients’ mental health, relieve their depressive symptoms, and prevent risk of suicide.

While this patient believed taking antidepressants helped manage their anxiety and depression, other patients demonstrated a lack of understanding of depression, their respective depression treatment, and its intended purpose and effect. Patients expressed a reverence for the medical field, often stating that they believed their treatment worked simply because the doctors told them it would. Finally, both Friendship Bench counselors and ART providers recognized that depressed patients may struggle to accept their HIV diagnosis and to take their ARTs consistently. For example, a Friendship Bench counselor and an ART provider commented

*Because of the counseling session, it helped the people … and now they are taking the medications consistently … this person is coming to collect drugs and their outlook [*toward their*] status has changed.*—Friendship Bench counselor

*Taking antidepressants has helped our friends with depression … to take their ARVs* [antiretrovirals] *adherently because when they are depressed they don’t even take the ARVs.* —ART provider

These descriptions demonstrate that clinic staff perceive that depression treatment supports ART adherence and engagement in HIV care.

### Sustainability

The clinical data suggest the program was not sustained. After enrollment into the program evaluation ended, the rate of screening dropped sharply (Table 5). By December 2019, 6 months after the study staff had complete data abstraction, ART providers had completely stopped screening patients newly initiating ART.

**TABLE 5.** Screening Rates During and After Enrollment Into the Program Evaluation

	PHQ-2 Screening,^a^ %	Complete PHQ-9 Screening,^b^ %
Program enrollment period (Apr. 2017–Nov. 2018)	91.8	90.5
Brief follow-up period (Feb. 2019–May 2019)	25.1	58.8

Abbreviations: PHQ-2, Patient Health Questionnaire-2; PHQ-9, Patient Health Questionnaire-9.

^a^ Among all new antiretroviral therapy initiators.

^b^ Among those who endorse the PHQ-2.

The clinical data suggest the program was not sustained and were partially in contrast with staff attitudes toward sustainability.

These quantitative findings are partially in contrast with staff attitudes toward sustainability identified in the qualitative interviews. The staff generally expressed a belief that they would continue to screen and manage depression after the study staff left the facilities. As heard from one ART provider,

*It will continue, because it’s our job as health workers to assist those people, because if we don’t assist, then who will? It’s like most people will not be taking the ARVs.* —ART provider

In fact, only 2 ART providers directly expressed any skepticism toward the sustainability of the program:

*Because there are people who are supporting [us] but if they can move out, it is when the true picture of their [providers’] attitude can be seen.* —ART provider

These ART providers acknowledged that the clinics receive a lot of support from the study staff and that challenges to sustainability may become evident after they leave. However, clinic staff and leadership did suggest certain resources would be key to supporting the sustainability of the program. These included increasing the number of trained staff, particularly Friendship Bench providers; offering refresher trainings and opportunities for continued learning; providing ongoing supervision; maintaining the stock of antidepressants; and having space for providing screening and counseling.

## DISCUSSION

This mixed-methods evaluation of an integrated depression treatment program found that, while early stages of training, depression screening, diagnosis, and treatment initiation were successfully integrated,^17^ treatment was not delivered as intended over time, clinic staff had mixed attitudes regarding the acceptability of the program, and sustainability was lacking. Over time, both ART providers and Friendship Bench counselors struggled to provide continued depression treatment owing to difficulties identifying and reassessing patients, negative attitudes, lack of integration into the EMR system, medication stock-outs, staff and space availability, cost of transport, and generally low retention. Further, program implementation relied heavily on study staff. While the quantitative program evaluation did not find that program exposure improved HIV care or depression outcomes,^20^ interviewed patients and clinic staff generally found the depression treatment both acceptable and helpful. The program was not sustained after the evaluation concluded, potentially because of limited staff, training, infrastructure, and supply of antidepressants.

Early stages of training, depression screening, diagnosis, and treatment initiation were successfully integrated; however, problems were apparent in the program delivery over time.

During the study, clinics failed to deliver Friendship Bench therapy with fidelity to the program protocol, particularly in regards to the low frequency of attended sessions over the 6-month duration of follow-up.^35^ The original Friendship Bench therapy protocol ideally called for 6 weekly therapy sessions, although patients were meant to set their own follow-up appointment dates. Similar to our findings, various evaluations of the original Friendship Bench therapy program in Zimbabwe suggest low patient engagement may not be uncommon. The pilot evaluation found that only 30% of participants completed 6 sessions in the first 6 weeks,^14^ the randomized control trial found around 40% completed 6 sessions over a 6-month period,^12^ and a mixed-methods longitudinal study over a 5-year period found nearly half of patients only attended 1 session, with only around 6% receiving 4 or more sessions.^36^ We found that scheduling weekly appointments was challenging for patients, often because of the prohibitive cost of transport and time spent at the clinic. Patient follow-up was also difficult for providers, in light of limited resources and challenges in contacting patients. The mixed-methods evaluation of the Friendship Bench in Zimbabwe also noted the lower-than-expected appointment attendance and follow-up challenges, which were attributed to the lack of incentives for Friendship Bench counselors to follow up with patients.^12^ While the Friendship Bench is a promising approach to providing evidence-based psychosocial therapy, further investigation of how best to engage patients over time is needed.

Providers managed to accurately identify patients with moderate to severe depression and started them on the correct dose of antidepressants, but fidelity to the MBC protocol over time was hampered by stock-outs, ART providers’ level of comfort with fluoxetine and increasing dosages, difficulties identifying depressed patients, lack of integration into the EMR system, and negative attitudes. These findings support previous conclusions around the feasibility of integrating screening and treatment initiation into ART.^17^^,^^20^ However, the challenges related to the provision of care over time are unsurprising because the providers are already overburdened and were expected to engage with the depression treatment program without additional incentives. A literature review on health systems facilitators and barriers to integration of HIV and chronic disease services found that successful integration programs have adequate, appropriately skilled, incentivized health care workers; supportive institutional structures; dedicated resources; and strong leadership and political will or support for the program.^37^ However, ensuring such a comprehensively supportive environment is particularly challenging in resource-limited settings. Furthermore, very few studies have been conducted on task-shifting programs that use algorithm-guided protocols for antidepressant prescription in the region, and those that have been conducted often do not rely on existing staff or incentivize the staff involved in the research.^10^^,^^15^ Further research that seeks to evaluate and compare integrated models of care for the provision of antidepressants is urgently needed to ensure the mental health needs of people living with HIV are met.^39^

Designing implementation science studies to evaluate programs that can readily (and rapidly) be adopted and integrated into routine care in the public sector is challenging.^39^^,^^40^ To assess such a depression treatment intervention, our program was intentionally designed to rely almost entirely on existing clinical staff and systems. Similarly, the evaluation was deliberately designed to influence the provision of care as little as possible. However, the study staff likely significantly supported both patients and providers involved in the program. The program-employed Friendship Bench counselors provided a significant proportion of the therapy sessions, suggesting a lack of effective task-shifting to existing community health workers. The study coordinator supervised and engaged clinic staff and assisted in supply-chain management to ensure the availability of antidepressants. The evaluation staff may have acted as “expert peers” by helping patients navigate clinical care, likely as a result of engaging patients in the consent process.^17^ Further, study staff helped providers identify depressed patients returning for care, thus artificially inflating the number of patients that would have been reassessed or would receive ongoing depression treatment. The lack of sustainability further suggests that the study staff were integral to the program implementation. Implementation science studies that support program implementation and program evaluation may need to take into account how the relationship between the evaluating team and the clinic staff may affect the implementation of the study itself.

Several key recommendations have emerged from this experience. As implemented, depression screening, diagnosis, and treatment initiation were successfully integrated into HIV care^17^; however, integrating follow-up treatment into HIV care in this setting was not feasible. Some of the implementation challenges appeared to be more immediately remediable and could be addressed through simple solutions such as the following:

- HTC counselors could administer the entire PHQ-9 at ART initiation. This would partially address the added burden of administering the PHQ-9 at ART initiation placed on ART providers, who are still well-positioned to determine a final diagnosis and provide appropriate treatment.
- The facility pharmacists could ensure an adequate supply of antidepressants through improved communication and coordination with central medical stores. This would allow providers to defer the choice of medication to the informed patient and prevent unnecessary and inappropriate switching of antidepressants.
- Friendship Bench appointments could be routinely scheduled on the same day as ART follow-up visits to alleviate the patient-side travel and resource burden and also allow the ART providers to consistently reconnect patients in Friendship Bench therapy to the counselors.

Some of the implementation challenges appeared to be more immediately remediable and could be addressed through simple solutions.

Resolving other challenges would require a fundamental change in the program design or substantially more resources than are currently available in the Malawian public health system. Nevertheless, the clinics would have benefitted from substantially more support, such as the following potential facilitators:

- The presence of a salaried, on-site coordinator or champion employed by the Ministry of Health would increase the likelihood of more faithful implementation. Such an individual would be well-positioned to foster clinic ownership of the program and provide the ongoing supervision and training that are needed to effectively build and sustain mental health care capacity.
- Integration into the EMR system is critical. This integration would help ensure that screening is completed and that returning patients are re-assessed and reinforce mental health capacity building and intervention delivery with fidelity.
- Finally, successful implementation requires a dedicated, clinic-based Friendship Bench counselor. Task-shifting to an already overburdened cadre of community health workers is an ineffective means of providing depression services.

### Limitations

Findings from this evaluation should be considered in light of limitations due to the program and evaluation design. First, neither the program nor the evaluation design was guided by a formal implementation science framework. However, both were designed in congruence with the key principles of implementation science, using methods that would promote the systematic uptake of evidence-based practices (e.g. the Friendship Bench and algorithm-based care) into routine care.^41^^,^^42^ We drew from various implementation science frameworks for this process evaluation to help ground our mixed-methods approach in implementation science theory.^21^^,^^35^ A small convenience sample of health care facility staff and patients from the program sites participated in the interviews. Notably, patients were still engaging with HIV care and may have had different opinions about the program than those lost to follow-up. Thus, the attitudes captured here may not be generalizable to all the patients exposed to the program.

In addition, all the patient participants were from Clinic A owing to the small number of individuals with depression identified at Clinic B after the launch of the intervention. While the shorter study period at Clinic B is largely responsible for the small number of depressed individuals, Clinic B also had a slightly lower prevalence of depression than Clinic A. Given the generally low retention rates, the study team decided to sample patients from Clinic A to achieve the target qualitative sample within the study period. However, this sampling decision does raise the potential for sampling bias, and it limited our ability to explore potential facility-level characteristics that could have been associated with the implementation success or failure.

Further, as the main objective of the study was to evaluate a program at the sites where participants worked or received care, it is possible that participants’ responses were subject to social desirability bias. The use of mixed-methods approaches is particularly useful for evaluating implementation outcomes,^21^ and in this case, the qualitative interviews helped to contextualize and provide nuanced information around the program’s shortcomings.

## CONCLUSION

This mixed-methods evaluation of a depression treatment program using antidepressants and psychotherapy in HIV clinics in Malawi found that although the early stages of integration and treatment initiation were successful,^17^ follow-up care was not delivered as intended, clinic staff had mixed attitudes regarding the acceptability of the program, and the program was not sustained. While these challenges may be responsible for the program’s lack of impact on retention, viral suppression, and depression remission,^20^ even the suboptimal depression treatment was appreciated by both patients and providers. This experience suggests that, while integrating depression screening, diagnosis, and treatment initiation in HIV care appeared feasible,^18^ without substantial support to supervise the implementation of such programs, continue to build and maintain the capacity of providers, integrate the program into the electronic medical records system and ensure the availability of psychotherapy counselors, the depression management aspects of this program may not be entirely appropriate or feasible in this setting. This process evaluation helps explain the shortcomings of the program implementation and provides valuable insight into how it could be improved. Further, similar mixed-methods exercises could be incorporated into more routine quality-improvement activities and offer potential insights into patient-, provider-, and system-level challenges to the delivery of evidence-based interventions. Moving the field of implementation science forward, we recommend that a conscious effort be made in study and program design to distance evaluation staff from the program implementation itself. Further research is also needed to test enhanced implementation strategies for integrating evidence-based mental health interventions into existing health care systems, particularly in low-resource settings.

## Supplementary Material

20-00607-Stockton-Supplement.pdf

## Notes

**Peer Reviewed**

**First published online:** August 4, 2021.
