# Supplementary material for: A Mixed-Methods Process Evaluation: Integrating Depression Treatment Into HIV Care in Malawi
Source: Glob Health Sci Pract. 2021 Sep 30;9(3):611–25. doi: 10.9745/GHSP-D-20-00607 (PMC8514021; doi:10.9745/GHSP-D-20-00607)
Supplement: 20-00607-Stockton-Supplement.pdf [file 20-00607-Stockton-Supplement.pdf]

POSTER- Depression Treatment with Antidepressants

Choosing a Depression Treatment Plan

PHQ-9 Total Score	Interpretation	Depression Treatment Plan
0-4	No depression	None
5-9	Mild depression	Friendship Bench
10-27	Moderate to severe depression	Start antidepressant

Starting an Antidepressant

PHQ-9 ≥ 10

↓

Confirm depression
Rule out bipolar and psychotic disorders

↓

Patient Education
Choose antidepressant with patient:
Amitriptyline or Fluoxetine

↓

Start antidepressant at Dosing Level 1
(AMI 50mg OD or FLU 20mg every other day)

Antidepressant Dosing Levels

	**Amitriptyline**	**Fluoxetine**
**Dosing Level 1** Initial Dose	50mg once daily at night	20mg on alternate days
**Dosing Level 2**	75mg once daily at night	20mg once daily
**Dosing Level 3**	100mg once daily at night	40 mg once daily
**If patient is still depressed at Dosing Level 3, contact consulting psychiatrist**		

Follow-up: Months 1 and 2

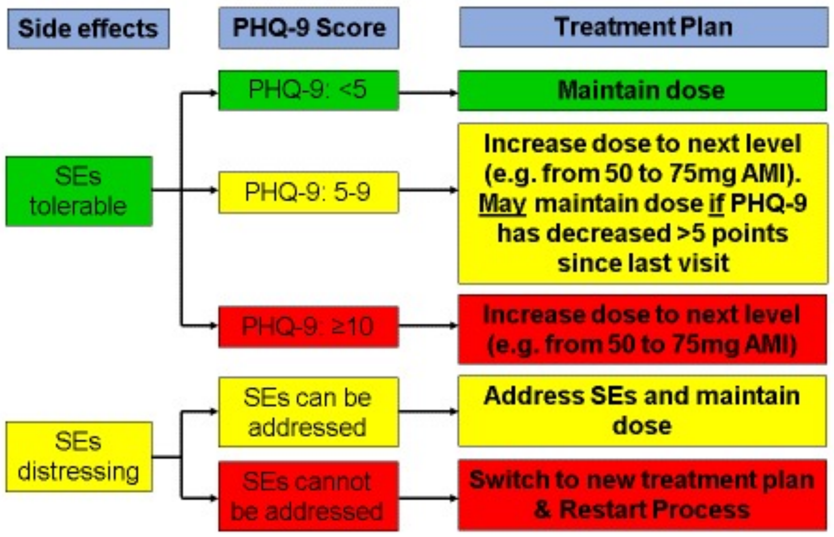

Follow-up: Month 3

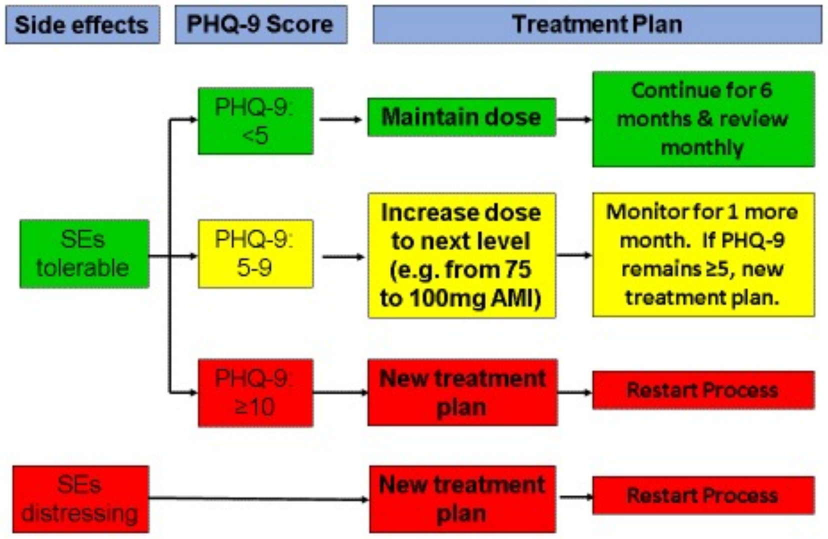

Alternate Treatment Plans

**If side effects cannot be addressed:**	Consider:<br>• **Switching** to different antidepressant<br>• **Switching** to Friendship Bench<br>• **Contact** consulting psychiatrist
If side effects are tolerable but patient remains depressed:	Consider:<br>• **Adding** Friendship Bench<br>• **Switching** to different antidepressant<br>• **Contact** consulting psychiatrist
